# The role of OSMRβ in inflammatory skin diseases

**DOI:** 10.3389/fimmu.2026.1931106

**Published:** 2026-10-09

**Authors:** Qianqian Zhang, Guifang Qin, Xiaomin Huang, Zudong Meng, Yulin Zou

**Affiliations:** 1 Department of Dermatology, Renmin Hospital, Hubei University of Medicine, Shiyan, China; 2 Department of Dermatology, Venereology and Allergology, University Medical Center Göttingen, Göttingen, Germany

**Keywords:** cytokine signaling, inflammatory skin diseases, OSMRβ, pruritus, targeted therapy

## Abstract

OSMRβ is a member of the IL-6 cytokine receptor family and plays a critical role in pruritus and immune-mediated skin diseases. Upon inflammatory stimulation, skin immune cells release OSM and IL-31. These cytokines signal through the heterodimeric receptor complexes OSMRβ/gp130 (type II OSM receptor) and OSMRβ/IL-31RA (IL-31 receptor), which are expressed on keratinocytes, fibroblasts, and dorsal root ganglion (DRG) neurons. Engagement of these receptors activates downstream JAK/STAT, MAPK/ERK and PI3K/AKT signaling cascades, leading to itch and inflammation that contributing to the pathogenesis of atopic dermatitis (AD), psoriasis (PSO), prurigo nodularis (PN) and familial primary localized cutaneous amyloidosis (FPLCA). Although a wide range of therapeutic options exist for these diseases, specific targeted treatments remain lacking. Vixarelimab, an OSMRβ-targeting monoclonal antibody, is currently under clinical evaluation for PN and AD.

## 1 Introduction

Inflammatory skin diseases comprise a heterogeneous group of chronic immune-mediated disorders characterized by recurrent inflammation, pruritus, skin damage, and pain (1, 2). Major examples include atopic dermatitis (AD), psoriasis (PSO), prurigo nodularis (PN), and familial primary localized cutaneous amyloidosis (FPLCA), among others, with substantial variation in their epidemiological patterns. These disorders impose a substantial burden on patients’ quality of life, manifesting as pruritus, pain, sleep disturbances, and associated psychiatric comorbidities (3–5). Moreover, AD frequently coexists with other atopic conditions such as asthma, allergic rhinitis, and food allergy (6, 7), while psoriasis is linked to metabolic syndrome, cardiovascular disease, and psoriatic arthritis (8). In patients with PN, psychiatric disorders including depression, anxiety, and obsessive–compulsive disorder are commonly observed (9). At the pathophysiological level, these diseases share several common features: impaired skin barrier function, aberrant TH2/TH17/TH22 immune responses, inflammatory cell infiltration, keratinocyte activation, and the perpetuation of a chronic itch–scratch cycle (8, 10–13).

Oncostatin M receptor β (OSMRβ) is a receptor subunit of the IL-6 cytokine receptor family that binds interleukin-31 (IL-31) and oncostatin M (OSM) and plays a critical role in skin immunity (14). IL-31 is a known pruritogenic factor in AD (15), whereas OSM is a pleiotropic cytokine that exerts important effects on inflammation, pruritus and the release of pro-inflammatory mediators in inflamed skin (5). Multiple studies have demonstrated that OSMRβ is upregulated in keratinocytes, fibroblasts, dorsal root ganglion (DRG) neurons and epithelial cells (16). These findings suggest that both OSM and IL-31 act through this receptor on skin-resident cells and sensory neurons to modulate inflammation and itch (**Figure 1**) (17).

**Figure 1:**
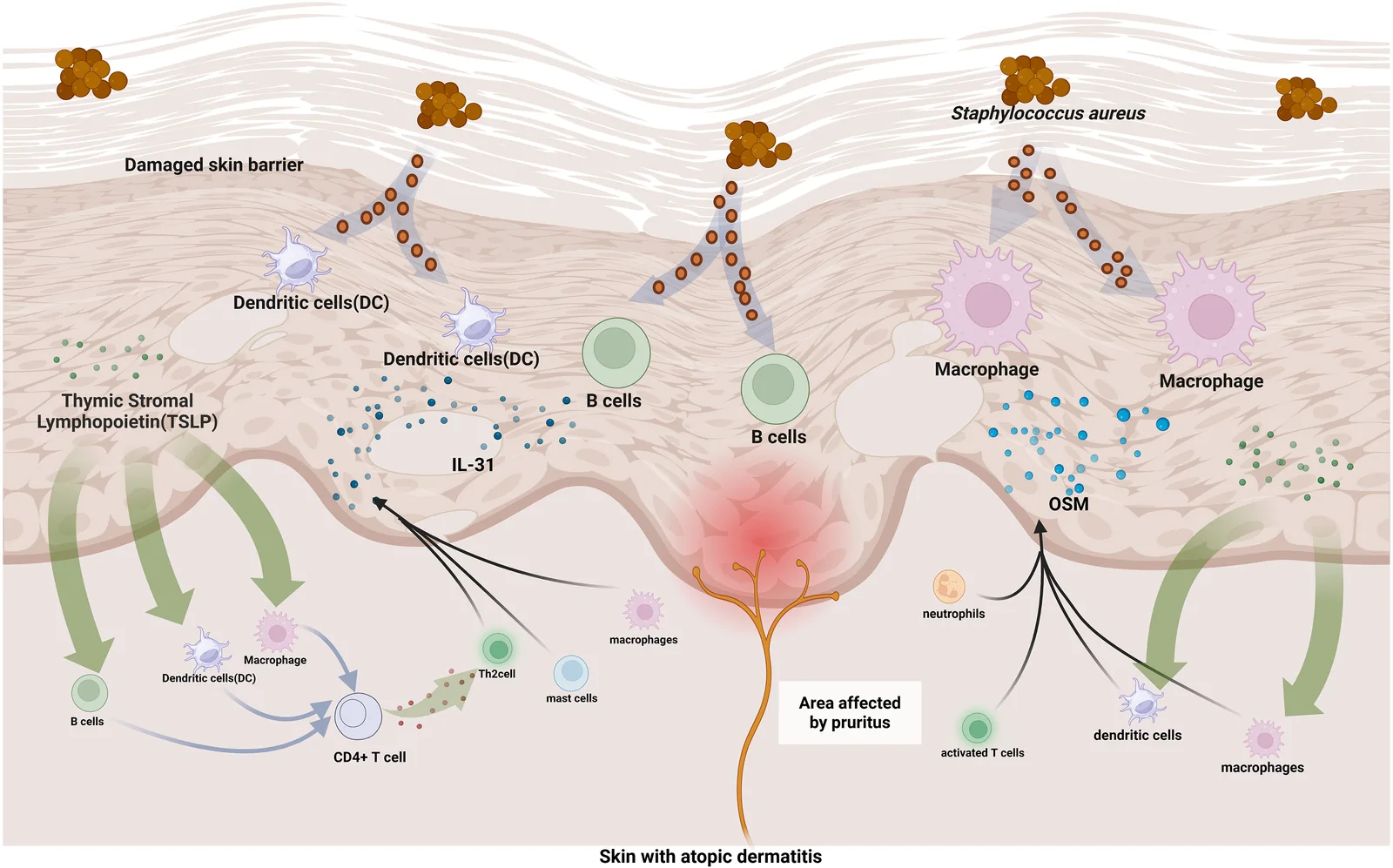
Following disruption of the stratum corneum barrier, allergens or pathogens (e.g., *Staphylococcus aureus*) penetrate the epidermis and activate antigen-presenting cells (monocytes/macrophages, B cells, and dendritic cells), which in turn stimulate thymic stromal lymphopoietin (TSLP) production. TSLP subsequently activates multiple immune cell types, including dendritic cells, T cells, and mast cells, leading to CD4^+^ T cell activation. Activated CD4^+^ T cells promote Th2-type cytokine secretion. Concurrently, Type 2 helper T (Th2) cells, mast cells, and macrophages produce IL-31, which drives type 2 inflammation and pruritus. In parallel, activated T cells, macrophages, dendritic cells, and neutrophils secrete Oncostatin M (OSM), promoting inflammatory responses, epidermal remodeling, and tissue damage.

Consequently, OSMRβ emerges as a pivotal hub connecting cutaneous inflammation with neurogenic pruritus. Boniface et al. reported elevated OSMRβ expression in psoriatic and AD lesions, where increased OSMRβ activity promotes pro-inflammatory and innate immune responses (18); other studies have also found increased numbers of OSMRβ-positive cells in the dermis of patients with PN (19). Furthermore, Ming-Wei Lin et al. reported that mutations in the OSMRβ gene may be implicated in the pathogenesis of primary cutaneous amyloidosis (PCA) (20). Therefore, this review summarizes the expression patterns, downstream signaling pathways, and therapeutic potential of OSMRβ in inflammatory skin diseases to advance our understanding of its pathophysiological roles and provide a theoretical basis for the development of next-generation targeted therapies.

To facilitate evaluation of the translational relevance of the available evidence, studies were categorized according to the experimental system supporting each specific finding: Level I, evidence derived directly from human tissues or clinical samples; Level II, evidence obtained from *in vivo* animal models; and Level III, evidence derived from *in vitro* or ex vivo cellular and molecular experiments, including experiments using primary human cells. For studies incorporating multiple experimental systems, the evidence level was assigned separately to each corresponding finding.

## 2 Inflammatory cytokine and OSMRβ receptor signaling pathway

In inflammatory skin diseases, the barrier function of the stratum corneum is impaired, allowing allergens and pathogens to penetrate the epidermis. These stimuli activate antigen-presenting cells, including monocytes/macrophages, dendritic cells, eosinophils, and lymphocytes, ultimately leading to CD4^+^ T-cell activation (18). Activated CD4+ T cells promote the secretion of Th2- and Th17-type cytokines. Concurrently, Th2 cells, dendritic cells, and macrophages release OSM, an IL-6 family cytokine, together with IL-31 (18, 21, 22). IL-31 signals through a heterodimeric receptor complex consisting of IL-31RA and OSMRβ, which is abundantly expressed in human dorsal root ganglia (DRG). Activation of this receptor on C-fiber nerve endings is thought to stimulate DRG neurons and induce pruritus (21, 23). In contrast, OSM regulates inflammatory responses through OSMRβ, the receptor subunit shared by both OSM and IL-31. Normal human epidermal keratinocytes express functional type II OSM receptors (comprising gp130 and OSMRβ), and OSM synergizes with TNF-α, IL-1, IL-17 and IL-22 to induce the expression of S100A7 and β-defensin 2 (14, 18).

Although IL-31 and OSM share OSMRβ and activate partially overlapping downstream signaling pathways, including JAK/STAT, PI3K/AKT, and ERK/MAPK, they differ in receptor composition, major cellular targets, and biological functions (23–27). IL-31 is predominantly produced by Th2 cells, mast cells and macrophages (28), and signals through the heterodimeric IL-31RA/OSMRβ receptor complex (29). Functionally, IL-31 is primarily associated with pruritic signaling, particularly through its actions on sensory neurons, while also regulating keratinocyte function (30, 31). In contrast, OSM is a pleiotropic pro-inflammatory cytokine generated by activated T cells, monocytes/macrophages, dendritic cells and neutrophils and signals through the type II OSM receptor composed of gp130 and OSMRβ (23, 26, 32). OSM acts on a broad range of cellular targets, including keratinocytes, fibroblasts, immune cells, and sensory neurons, thereby contributing to cutaneous inflammation, tissue remodeling, and sensory-neuron sensitization (18, 33, 34). Thus, despite sharing OSMRβ and partially overlapping downstream pathways, the distinct receptor complexes and cellular targets of IL-31 and OSM result in divergent biological effects, with IL-31 being more closely associated with direct pruritic signaling and OSM exerting broader inflammatory, tissue-remodeling, and neuromodulatory effects (30, 34) (**Table 1**).

**Table 1:** Evidence for the upregulation of OSMR in inflammatory skin diseases.

Disease	OSMR/OSMRβ expression	Type of evidence	Quantification criteria/threshold	Sample source/model	Reference
Atopic dermatitis (AD)	Type II OSMR expressed in AD lesions; enhanced OSM transcripts	Tissue samples (bulk transcriptome, RT-PCR, microarray) (Level I)	-dCt(log2 scale)or2^ΔΔCt/ P<0.05	Human AD lesions vs healthy skin	(18)
Atopic dermatitis (AD)	IL-31RA and OSMR expressed in keratinocytes; upregulated by TLR-2 ligand and IFN-γ	Cellular assays (mRNA and protein levels) (Level III)	MFI or RFI P< 0.05	Human primary keratinocytes (HPKs)	(29)
Atopic dermatitis (AD)	OSMRβ functional involvement in AD model mice	Animal model (AD mouse model) (Level I)	Skin score 0–18/Scratching episodes per 2 hours/P< 0.05	AD mouse model (OSM treatment/anti-OSMRβ antibody intervention)	(14)
Atopic dermatitis (AD)	OSMRβ overexpression in DRG cell line	Tissue samples(Level I)	-dCt(log2 scale)or2^ΔΔCt/P<0.05	AD model mice	(55)
Psoriasis (PSO)	Type II OSMR expressed in psoriatic lesions; enhanced OSM transcripts	Tissue samples (bulk transcriptome, RT-PCR, microarray) (Level I)	-dCt(log2 scale)or2^ΔΔCt/P<0.05	Human psoriatic lesions vs healthy skin	(18)
Psoriasis (PSO)	OSM highly upregulated; OSMR expressed in pruritus-specific natriuretic peptide B (Nppb) neurons	Single-cell sequencing, tissue samples (Level I/Level III)	TPM, log2FC P< 0.05	Human psoriatic lesions, mouse DRG neurons	(34)
Prurigo nodularis (PN)	Increased dermal OSMRβ^+^ cells in PN	Tissue samples (immunofluorescence staining) (Level I)	Cell density/intensity grading/P< 0.05	Human PN lesions	(19)
Prurigo nodularis (PN)	Significantly elevated mRNA and protein levels of OSMRβ, IL-31, IL-31RA and OSM in PN lesions	Tissue samples (immunohistochemistry, mRNA analysis) (Level I)	-dCt(log2 scale) or2^ΔΔCt/ P<0.05/Cell density/intensity grading	Human PN lesions vs non-lesional skin	(56)
Familial primary localized cutaneous amyloidosis (FPLCA)	Missense mutations in OSMR gene (genetic evidence)	Genetic analysis (candidate gene analysis) (Level I)	optical density ratio(target/internal control)/P < 0.05	Human FPLCA pedigrees	(45)
Familial primary localized cutaneous amyloidosis (FPLCA)	Loss-of-function OSMR mutations enhance basal keratinocyte differentiation via the OSMR/STAT5/KLF7 axis	Cellular assays, animal models (gene-edited mice), 3D human epidermal culture(Level I/Level II/Level III)	optical density ratio/P < 0.05	PLCA patient keratinocytes, HaCaT cells, NHEKs, OSMR-knockout mice	(81)
Primary cutaneous amyloidosis (PCA)	PCA can be caused by mutations in either OSMR or IL31RA genes	Genetic analysis(Level I)	mutation frequency/LOD score/LOD score > 3/P < 0.01	Human PCA pedigrees	(20)
Post-burn hypertrophic scars	Elevated expression levels of IL-31, IL-31RA and OSMR in hypertrophic scars	Tissue samples (RT-PCR, western blot) (Level I)	Pearson or Spearman rho, p < 0.05	Human post-burn hypertrophic scars vs normal tissue	(16)

JAK/STAT signaling represents a major downstream pathway shared by both cytokine-receptor complexes (35). Ligand binding activates receptor-associated JAKs, leading to phosphorylation and nuclear translocation of STAT proteins (26, 36–38). However, the magnitude and pattern of STAT activation differ between IL-31 and OSM and are cell-context dependent. In normal human primary keratinocytes, IL-31 predominantly induces STAT3 phosphorylation (Level III) (29), whereas OSM induces robust STAT3 and STAT1 phosphorylation in human epidermal keratinocytes (Level III) (39). A similar distinction has been observed in dermal fibroblasts, in which OSM markedly activates STAT3 and STAT1, whereas IL-31 induces only minimal STAT3 activation and fails to induce MCP-1 expression (Level III) (39). Beyond JAK/STAT signaling, both receptor systems can engage MAPK/ERK and PI3K/AKT pathways, although their relative activation appears to depend on ligand and cellular context.

The MAPK/ERK pathway represents another important downstream branch of OSMRβ signaling (37, 40–42). OSM binding to the type II OSM receptor induces ERK1/2 phosphorylation. Recent studies have shown that OSM upregulates CD73 expression in keratinocytes through the MAPK pathway. This promotes an adenosine-dependent anti-inflammatory response, suggesting that OSM/MAPK signaling may exert both pro-inflammatory and compensatory anti-inflammatory effects in psoriasis (43).

The PI3K/AKT pathway is the third major downstream signaling cascade mediated by OSMRβ (44). IL-31 activates PI3K/AKT through the OSMRβ/IL-31RA heterodimeric receptor complex (41, 42, 44). In keratinocytes stimulated with the M5 cytokine cocktail, the PI3K/AKT pathway is robustly activated (43)(Level III).In keratinocytes derived from patients with familial primary localized cutaneous amyloidosis (FPLCA), immunoblot analysis of phosphorylated STATs, ERK1/2, and AKT demonstrated that OSM stimulation induced phosphorylation of these signaling molecules, whereas IL-31 failed to elicit detectable phosphorylation (Level III). The phosphorylation levels induced by OSM were approximately 65–95% lower in FPLCA keratinocytes than in control keratinocytes, as determined by densitometric analysis. Nevertheless, both FPLCA and control keratinocytes exhibited dose-dependent responses to OSM. In contrast, IL-6, whose receptor complex does not contain OSMRβ, failed to alter phosphorylation in FPLCA keratinocytes (45). These differential responses further illustrate that sharing OSMRβ does not imply identical signaling capacity for OSM and IL-31; rather, ligand-specific receptor composition and cellular context critically shape downstream signaling.

In keratinocytes, OSMRβ activation coordinately engages the JAK/STAT, MAPK/ERK, and PI3K/AKT signaling pathways, leading to the production of pro-inflammatory cytokines (IL-1β, IL-6, and IL-8) and antimicrobial peptides (S100A7, S100A9, and β-defensin 2) (18, 44), while promoting keratinocyte proliferation and survival. In sensory neurons, IL-31 signals through the OSMRβ/IL-31RA receptor complex to activate the JAK/STAT pathway, particularly STAT3. The IL-31 receptor complex is highly expressed in human DRG, supporting its role in itch transmission. In addition, IL-31 induces the expression of the chemokines CCL1, CCL17, and CCL22, which recruit T cells to the affected skin. These infiltrating T cells subsequently produce additional IL-31, thereby establishing a positive feedback loop (**Figure 2**) (21).

**Figure 2:**
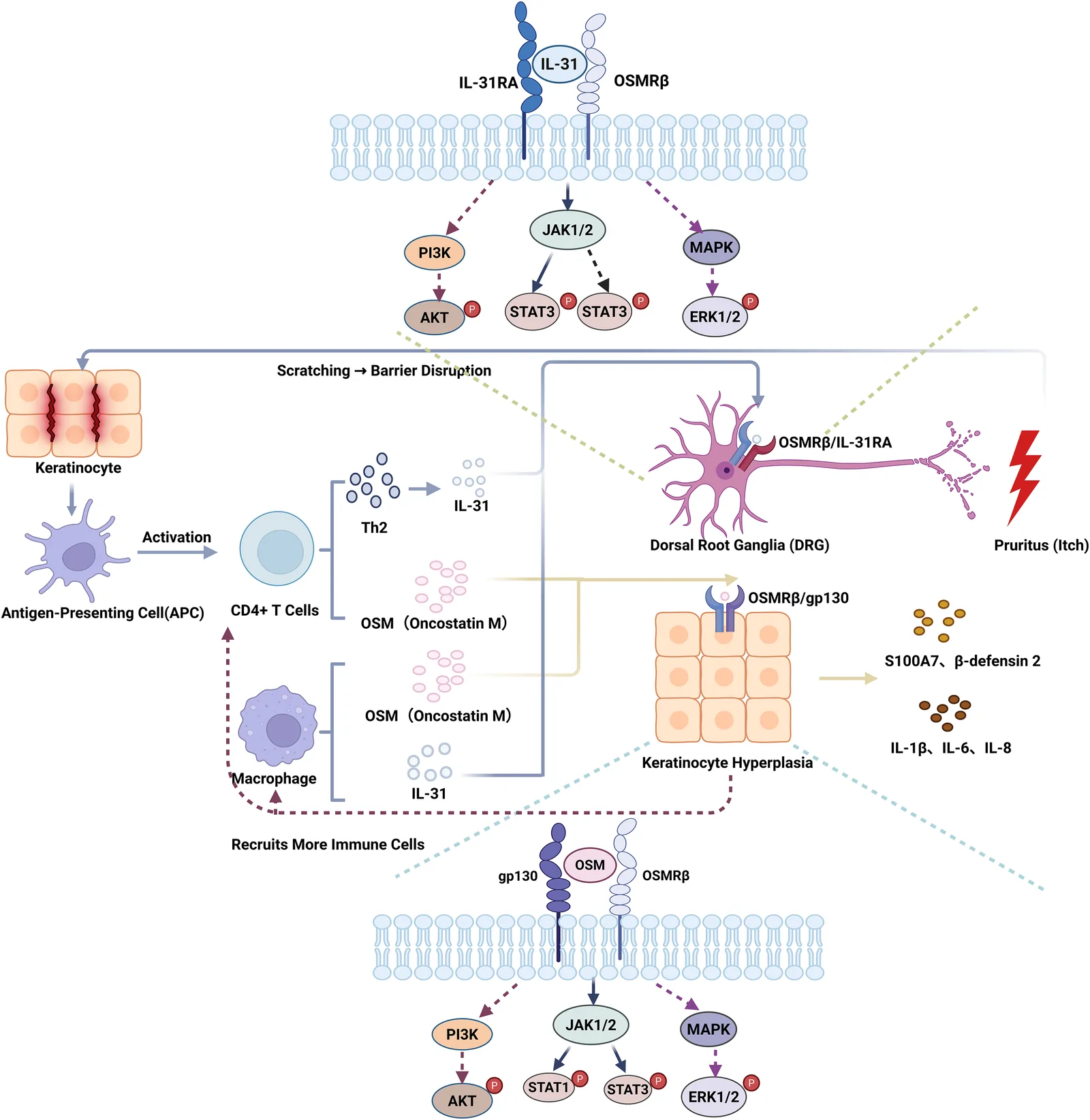
Skin barrier disruption activates antigen-presenting cells (APCs), leading to the activation of CD4^+^ T cells and macrophages. T helper 2 (Th2) cells predominantly produce IL-31, while OSM is produced by activated immune cells, including T cells and macrophages. Macrophages may also contribute to IL-31 production, further amplifying cutaneous inflammation. IL-31 binds to the IL-31RA/OSMRβ receptor complex expressed on dorsal root ganglion (DRG) sensory neurons and activates downstream JAK/STAT, PI3K/AKT, and MAPK/ERK signaling pathways, thereby promoting pruritus. Subsequent scratching aggravates epidermal barrier disruption and contributes to the itch–scratch cycle. In parallel, OSM binds to the OSMRβ/gp130 receptor complex on keratinocytes and activates overlapping downstream signaling pathways, promoting keratinocyte hyperplasia, epidermal remodeling, and the production of inflammatory mediators, including S100A7, β-defensin 2, IL-1β, IL-6, and IL-8. These mediators may further recruit and activate immune cells, thereby sustaining cutaneous inflammation. IL-31 is closely associated with pruritic signaling, whereas OSM has prominent effects on keratinocyte activation, epidermal remodeling, and inflammation. Solid arrows indicate well-established signaling or functional relationships, whereas dashed arrows indicate context-dependent signaling relationships.

## 3 Specific roles of OSMRβ in different inflammatory dermatoses

### 3.1 Atopic dermatitis

AD is a chronic inflammatory skin disease characterized by intense pruritus and eczematous lesions. OSMRβ, the shared receptor subunit for OSM and IL-31, has been implicated in both pruritic and inflammatory processes in AD (14). Accordingly, available evidence suggests that IL-31 signals through the IL-31RA/OSMRβ heterodimeric receptor to induce pruritus in AD (21)(Level I). However, although IL-31 contributes to both itch and skin lesion development, selective blockade of IL-31 alone does not significantly improve cutaneous lesions, indicating that additional OSMRβ ligands, particularly OSM, also contribute to skin inflammation (14).

In an AD mouse model, Komori et al. used 8-week-old male mice and randomly assigned animals with comparable disease severity scores (7–11; mean scores 8.50 ± 1.69 vs. 8.33 ± 1.50) to different treatment groups. OSM administration exacerbated skin lesions and increased IL-4 levels in the draining lymph nodes (14)(Level II). At the cellular level, normal human epidermal keratinocytes express functional type II OSM receptors composed of gp130 and OSMRβ, which are also expressed in AD and psoriatic lesions. Through activation of the STAT3 and MAPK signaling pathways, OSM induces a pro-inflammatory gene expression program in keratinocytes, thereby promoting innate and inflammatory immune responses (18). Collectively, these experimental findings support a potential role for OSM/OSMRβ signaling in cutaneous inflammation associated with AD, possibly through effects on keratinocytes and Th2-associated immune responses. However, the extent to which these mechanisms operate in human AD requires further validation.

### 3.2 Psoriasis

PSO is a common inflammatory skin disease characterized by epidermal hyperplasia and chronic inflammation (46–51). Previous studies have demonstrated that OSM and the OSMRβ/gp130 heterodimeric complex are highly expressed in psoriatic lesions (18, 52)(Level I). OSM is a potent activator of keratinocytes and acts synergistically with TNF-α, IL-1, IL-17, and IL-22 to induce the expression of S100A7 and β-defensin 2, two characteristic markers of psoriatic skin (18)(Level III). *In vitro*, OSM has been shown to promote keratinocyte proliferation and survival through ERK- and STAT3-associated upregulation of the anti-apoptotic protein Livin56 (53)(Level III). Keratinocytes stimulated with the M5 cytokine cocktail, which mimics the psoriatic inflammatory microenvironment, produce pro-inflammatory cytokines, including IL-1β, IL-6, and IL-8, as well as the antimicrobial peptide S100A9. This stimulation also activates multiple downstream signaling pathways, including JAK/STAT, MAPK/ERK, and PI3K/AKT (43) (Level III).

Beyond its potential contribution to cutaneous inflammation, emerging experimental evidence suggests that OSM/OSMRβ signaling may also contribute to pruritus through modulation of sensory-neuron responsiveness. Unlike classical pruritogens, OSM does not directly activate sensory neurons but instead sensitizes them by enhancing their responsiveness to pruritic stimuli and increasing neuronal excitability, thereby amplifying itch perception. Consistent with this mechanism, sensory neuron-specific deletion of Osmr markedly attenuated both OSM-induced itch sensitization and inflammatory itch in mice (54). Tseng et al. further demonstrated, using dissociated Trpv1-GCaMP6 DRG neurons stimulated with OSM, leukotriene C_4_ (LTC_4_), histamine, and capsaicin, that OSM failed to directly activate sensory neurons but significantly increased their responsiveness to pruritic stimuli. Because histamine was the only pruritogen evaluated in this study, it remains unclear whether other pruritogens similarly modulate OSM-sensitized pruriceptive neurons (34).

At the *in vivo* level, Tseng et al. generated DRG-specific Osmr conditional knockout (cKO) mice and demonstrated that sensory neuron-specific deletion of Osmr significantly attenuated both OSM-sensitized itch and inflammatory itch (Level II). The authors further used the gp130 inhibitor SC144 to block the OSMRβ/gp130 receptor complex (Level II). At the behavioral level, SC144 treatment almost completely abolished OSM-induced delayed scratching. However, these findings should be interpreted with caution. OSMR is expressed not only in sensory neurons but also in non-neuronal cells, including keratinocytes. In addition, SC144 inhibits gp130 signaling and is therefore not specific to the OSM/OSMRβ pathway. Because IL-6 also signals through gp130 and is upregulated in inflamed skin, some of the observed effects of SC144 may have been mediated through keratinocytes or other IL-6Rα-expressing non-Nppb neurons rather than sensory neurons alone (34).

Ishikawa et al. further demonstrated that OSM promotes neurite outgrowth in both primary mouse DRG neurons and a DRG-derived cell line. To assess dermal nerve elongation, the authors performed immunofluorescence staining using the pan-neuronal marker PGP9.5. However, this marker does not distinguish C-fiber subtypes (including NP1, NP2, and NP3) from other sensory nerve fibers (Level III). *In vivo*, subcutaneous administration of OSM significantly enhanced dermal nerve elongation (Level I). Nevertheless, whether OSM-induced inflammation directly contributes to itch was not examined (55).

Taken together, these complementary studies demonstrate that OSM sensitizes sensory neurons by enhancing their responsiveness to pruritic stimuli and increasing neuronal excitability. *In vitro* experiments permit direct assessment of neuronal electrophysiological responses and neurite outgrowth following OSM stimulation but cannot fully recapitulate the complex skin microenvironment, where keratinocytes and immune cells also express OSMRβ and influence OSM signaling. In contrast, *in vivo* studies enable evaluation of behavioral outcomes, such as scratching behavior, as well as histological changes, including nerve elongation and skin inflammation. However, interpretation of these findings is complicated by several factors, including the contribution of non-neuronal target cells, the lack of specificity of gp130 inhibition by SC144, and the inability of PGP9.5 staining to distinguish among sensory nerve fiber subtypes. Overall, the available evidence supports OSM as an important neuromodulator of itch and suggests that targeting OSMRβ may simultaneously suppress OSM-driven inflammation and pruritus, representing a promising therapeutic strategy for psoriasis.

Although the majority of current evidence supports a pro-inflammatory role for OSM in psoriasis by promoting keratinocyte proliferation, inflammatory mediator production, and immune cell recruitment, several studies have suggested that OSM may also exert compensatory anti-inflammatory effects through MAPK-dependent signaling and negative feedback mechanisms. These apparently contradictory findings are likely explained by context-dependent factors, including disease stage, cellular composition, receptor expression (OSMR versus leukemia inhibitory factor receptor, LIFR), and the surrounding cytokine milieu. Therefore, OSM signaling should be regarded as a dynamic regulator rather than a purely pro-inflammatory cytokine in psoriasis.

In summary, available experimental and human-tissue evidence implicates OSMRβ signaling in both inflammatory and pruritic processes relevant to psoriasis. OSM/OSMRβ signaling may contribute to cutaneous inflammation through effects on keratinocyte activation, inflammatory mediator production, and epidermal responses, whereas experimental neuronal studies suggest that OSM can enhance sensory-neuron responsiveness to pruritic stimuli. Further human studies are required to determine the relative contribution of these mechanisms to psoriasis-associated inflammation and pruritus.

### 3.3 Prurigo nodularis

Prurigo nodularis (PN) is a chronic inflammatory dermatosis characterized by intensely pruritic, hyperkeratotic nodules (19, 56). Its typical histopathological features include epidermal hyperplasia, dermal fibrosis and infiltration of type 2 inflammation-associated immune cells within the dermis. Within this pathological framework, OSM/OSMRβ signaling has been implicated in processes related to epidermal hyperplasia, inflammation, and fibrosis (57, 58).

Multiple studies have demonstrated that OSMRβ protein and mRNA expression are significantly upregulated in PN lesions compared with normal skin or non-lesional skin (56)(Level I). Within the proposed framework of the four major pathogenic elements of PN—type 2 inflammation, epidermal hyperplasia, dermal fibrosis, and neuroimmune pruritus—dermal fibrosis represents a central pathogenic feature alongside the other three (19). OSM/OSMRβ signaling, acting through dermal macrophages that express OSMRβ (which account for 51.8% of OSMRβ-positive cells), promotes their polarization toward a profibrotic phenotype, thereby driving extracellular matrix deposition and nodule formation (19). Hashimoto et al. further confirmed that the number of OSM-positive cells is significantly increased in PN lesions (p < 0.05), with T lymphocytes (49.8%) and mast cells (26.8%) representing the major cellular sources (19). Effects of OSM/OSMRβ signaling on fibroblasts and macrophages provide a potential mechanism through which this pathway may contribute to cutaneous inflammation and dermal fibrosis; however, its direct contribution to these pathological features in human PN remains incompletely defined (57).

Notably, OSMRβ protein is also expressed in dermal nerve fibers within PN lesions (56), suggesting a potential role in neural remodeling. Both IL-31 and OSM act on sensory neurons via the OSMRβ subunit shared by their respective receptor complexes: the IL-31/OSMRβ/IL-31RA axis directly triggers pruritic signals, whereas the OSM/OSMRβ/gp130 axis exerts a neuronal sensitizing effect, enhancing neuronal responsiveness to pruritogenic stimuli (34, 59). Hashimoto et al. found that itch intensity in PN patients correlates positively with the number of dermal IL-31-positive cells, OSM-positive cells and IL-31RA-positive cells (19)(Level I). However, although the number of OSMRβ-positive cells is significantly increased in PN lesions (p < 0.05), its correlation with itch intensity did not reach statistical significance (Spearman’s r = 0.375, p > 0.05) (19). These findings suggest that OSMRβ expression alone may not determine itch severity. Instead, OSMRβ may contribute primarily to OSM-mediated neuronal sensitization and fibrotic remodeling, although further mechanistic studies are required to clarify its specific role in PN pathogenesis.

### 3.4 Familial primary localized cutaneous amyloidosis

FPLCA is an autosomal dominant disorder characterized by chronic pruritus and the deposition of keratin filament-associated amyloid in the dermis. In 2008, Arita et al. identified heterozygous missense mutations in the OSMR gene through genetic analysis of three families with FPLCA(Level I). Family 1 carried a c.2072T→C transition (p.I691T), while families 2 and 3 shared a c.1853G→C substitution (p.G618A) (45). Both pathogenic amino acid replacements are located within the fibronectin type III-like (FNIII) domain of the OSMRβ extracellular region, a region previously shown to be essential for receptor function and signal transduction (60). Previous structure–function data on gp130 showed that deletions or single-amino-acid substitutions in the FNIII domain disrupt gp130 homodimerization or gp130–LIFR heterodimer formation (60, 61). Given that OSMRβ shares a similar domain organization with gp130, Arita et al. postulated that the p.G618A and p.I691T substitutions in the OSMRβ FNIII domain might interfere with proper receptor coupling of OSMRβ to gp130, as well as to IL-31RA (45). This hypothesis was subsequently validated in functional experiments: upon OSM stimulation, FPLCA keratinocytes exhibited a 65–95% reduction in phosphorylation of STATs, ERK and Akt; no phosphorylation was observed following IL-31 stimulation (45)(Level III). These findings confirmed that mutations in the OSMRβ FNIII domain indeed lead to attenuated signaling activation for both OSM and IL-31. The study by Arita et al. (45) established a genetic link between OSMR mutations and FPLCA. However, it did not directly connect the specific gene mutations, the cell-derived proteins (KRT5/14), and the histological changes (amyloid deposition). Building on this, Bourguiba et al. (62) combined LC-MS/MS, immunoelectron microscopy, and genetic analyses to provide further evidence linking OSMR mutations with dermal amyloid deposits derived from basal keratins KRT5 and KRT14 (62) (Level I).

Taken together, the pathogenesis of FPLCA can be summarized as follows. Mutations in OSMR impair OSM- and IL-31-mediated signaling in keratinocytes, leading to reduced activation of the JAK/STAT, MAPK/ERK, and PI3K/AKT pathways. Consequently, the diminished activation of these pro-survival pathways increases the susceptibility of keratinocytes to apoptosis. Persistent scratching secondary to chronic pruritus further exacerbates keratinocyte injury and apoptosis, resulting in the release of KRT5 and KRT14. These keratins subsequently aggregate within the dermis and ultimately form amyloid deposits.

## 4 Current therapeutic strategies for inflammatory dermatoses

### 4.1 Atopic dermatitis

Current treatment for AD follows a stepwise approach, with therapy escalated according to disease severity. For mild-to-moderate disease, first-line treatment consists of basic skin care and topical anti-inflammatory agents. Patients with moderate-to-severe AD may require phototherapy, systemic immunosuppressants (including ciclosporin, methotrexate, and azathioprine), targeted biologics (such as dupilumab, tralokinumab, and lebrikizumab), or JAK inhibitors (63). However, conventional treatments are often associated with adverse events and limited efficacy; the response to standard regimens is variable and frequently insufficient, and many carry substantial side effects (64). For example, in a pivotal 16-week, randomized, double-blind phase 3 trial of dupilumab in adult patients with moderate-to-severe atopic dermatitis (IGA ≥ 3) whose disease was inadequately controlled by topical therapies, more than 60% of patients did not achieve clear or almost-clear skin at week 16 (65).

### 4.2 Psoriasis

The treatment of psoriasis has similarly evolved from conventional drugs to biologics (66). Biologics targeting key cytokines involved in psoriasis pathogenesis, particularly interleukin-23 (IL-23) and interleukin-17 (IL-17), have become first-line therapies for moderate-to-severe psoriasis and have substantially improved clinical outcomes (67). Nevertheless, conventional systemic agents (such as methotrexate, ciclosporin and acitretin) are still often preferred because of their oral administration and relatively lower cost; however, they are limited by treatment resistance or inefficacy in some patients, as well as high costs in certain settings (66). In addition, marked inter-individual variability in response and incomplete responses remain challenges in moderate-to-severe psoriasis (66).

### 4.3 Prurigo nodularis

PN remains a challenging disease to treat, with limited therapeutic options available. Conventional therapies are frequently associated with inadequate efficacy, safety concerns, and poor treatment responses. Consequently, there is an urgent need to develop novel therapeutic strategies for patients with moderate-to-severe PN. Dupilumab was the first biologic approved by both the European Medicines Agency (EMA) and the U.S. FDA for the treatment of moderate-to-severe chronic PN (68). More recently, nemolizumab, a humanized monoclonal antibody targeting IL-31RA, has also been approved for both AD and PN (69). Despite these advances, unmet therapeutic needs remain, highlighting the need for safer and more effective treatment options.

### 4.4 Primary cutaneous amyloidosis

Primary cutaneous amyloidosis (PCA) is a rare disorder characterized by amyloid deposition within the dermal papillae. Primary localized cutaneous amyloidosis (PLCA), the most common form of PCA, is confined to the skin and comprises several clinical subtypes, including FPLCA. The pathogenesis of PLCA is multifactorial and has been closely associated with mechanical factors such as friction, chronic pruritus, and persistent scratching. In contrast, FPLCA is primarily driven by genetic mutations, most notably in the OSMR gene (70). Owing to the rarity of this condition, standardized treatment strategies remain unavailable, and current therapeutic options are largely empirical. Although systemic retinoids and psoralen plus ultraviolet A (PUVA) therapy may provide symptomatic relief, their clinical use is limited by adverse effects, including teratogenicity and mucocutaneous toxicity. Moreover, many patients exhibit poor tolerance or an inadequate response to conventional treatments, such as topical corticosteroids and systemic retinoids (71).

## 5 Novel targeted therapeutics against OSMRβ

Based on the previously reviewed data, targeting OSMRb signagling may simultaneously modulate pruritus and TH2 inflammatory responses in the aforementioned disease states (13, 57).

Vixarelimab (KPL-716) is a fully human monoclonal antibody that targets the OSMRβ, thereby blocking both IL-31- and OSM-mediated signaling (69). In a phase 2b randomized clinical trial of vixarelimab in patients with moderate-to-severe PN, treatment with vixarelimab demonstrated rapid, sustained and dose-dependent clinical benefits, with a favorable safety profile (72). In a separate randomized, double-blind, placebo-controlled phase 2a study, vixarelimab achieved rapid itch reduction by week 8 in patients with moderate-to-severe PN, with one-third of patients achieving clear or almost-clear skin. Vixarelimab has completed phase 2a and phase 2b randomized clinical trials in patients with PN, demonstrating improvements in pruritus and skin lesions (73).

Regarding the safety profile of vixarelimab, a randomized, double-blind, placebo-controlled phase 2a trial in patients with moderate-to-severe PN demonstrated that the most common adverse events (incidence >5%) included upper respiratory tract infection, nasopharyngitis, headache, nummular eczema, urticaria, and procedural headache (74). Ständer et al. further reported in a subsequent phase 2b randomized clinical trial that the most common treatment-emergent adverse events (incidence ≥5%) in the vixarelimab group were headache (4.3%–8.5%), eczema (2.1%–8.5%), and fatigue (2.1%–10.6%), whereas in the placebo group, the most common treatment-emergent adverse events were COVID-19 (4 [8.3%]), insomnia (3 [6.3%]), and hypertension (3 [6.3%]). In addition, an analysis covering the period from the first quarter of 2024 to the second quarter of 2025 from the U.S. FDA Adverse Event Reporting System showed that the most frequently reported adverse events during long-term nemolizumab treatment were COVID-19, nasopharyngitis, atopic dermatitis, upper respiratory tract infection, headache, and asthma (75). Overall, the majority of adverse events reported in the above studies were mild to moderate in severity, and the rate of discontinuation due to adverse events was low.

Beyond Vixarelimab, other anti-OSMRβ antibodies have also shown therapeutic potential in preclinical settings. Komori et al. reported that treatment with an anti-OSMRβ monoclonal antibody (7D2) reduced skin severity scores in AD model mice (14). Moreover, 7D2 decreased scratching behavior, concomitant with a reduction in the number of OSMRβ-positive neurons in the dorsal root ganglia. 7D2 also lowered serum concentrations of IL-4, IL-13 and IgE, as well as gene expression levels of IL-4 and IL-13 in lymph nodes (14). These findings indicate that anti-OSMRβ monoclonal antibodies may emerge as novel drug candidates for AD.

Recently, Zhang et al. reported a monoclonal antibody (2O2) directed against the proximal extracellular domain of OSMRβ. Structural analyses revealed that 2O2 binds at an oblique angle relative to the cell membrane, generating steric hindrance and inducing local invagination of the lipid bilayer. Mechanistically, 2O2 inhibits IL-31-induced STAT5 phosphorylation through antibody-induced internalisation of OSMRβ from the cell surface, rather than via competitive ligand blockade (76). This mechanism enables the clearance of the entire signaling complex from the cell surface, which represents a functional advantage over conventional cytokine-blocking antibodies.

Although nemolizumab does not directly target OSMRβ, it is a monoclonal antibody directed against IL-31 receptor A (IL-31RA) that functions by blocking the upstream IL-31/OSMRβ signaling pathway (77). The U.S. FDA has approved nemolizumab for the treatment of both AD and PN (78, 79). In Japan, nemolizumab has been approved for AD-associated pruritus since 2022 and received additional approval for PN in 2024 (80).

Current clinical evidence suggests that receptor blockade with nemolizumab has demonstrated broader clinical efficacy than IL-31 ligand neutralization. Although both ligand- and receptor-targeting strategies are designed to inhibit IL-31 signaling, their mechanisms of action differ fundamentally. Anti-IL-31 antibodies neutralize the ligand before receptor engagement, whereas nemolizumab directly targets IL-31RA, thereby preventing IL-31 from activating the IL-31RA/OSMRβ heterodimeric receptor complex. Current clinical studies suggest that receptor blockade may achieve broader therapeutic efficacy than ligand neutralization, possibly because it provides more complete inhibition of IL-31RA/OSMRβ-mediated signaling. However, this interpretation should be made with caution, as evidence is derived from independent clinical trials rather than direct head-to-head comparisons, and further mechanistic and comparative studies are needed.

In summary, OSMRβ represents an attractive therapeutic target because its inhibition simultaneously blocks IL-31-mediated pruritus and OSM-mediated inflammation and fibrosis, offering broader therapeutic potential than strategies directed against a single cytokine. Anti-OSMRβ monoclonal antibodies, including vixarelimab, 7D2, and 2O2, therefore represent promising therapeutic candidates for AD and other chronic pruritic dermatoses (**Table 2**).

**Table 2:** Comparison of anti-OSMRβ antibodies: Vixarelimab, 7D2, and 2O2.

Feature	Vixarelimab	7D2	2O2
Targeted epitope	OSMRβ	OSMRβ	The membrane-proximal extracellular domain of canine OSMRβ (cOSMRβ)
Experimental models	Patients with moderate-to-severe prurigo nodularis (PN) (Level III)	Spontaneously occurring AD-like NC/Nga mice and mite-extract-induced AD-like NC/Nga mice(Level II)	DH-82 cells expressing cIL-31RA and cOSMRβ (*in vitro* and structural studies; no *in vivo* model described)(Level III)
Dosing regimen	Double-blind period: subcutaneous injection once every 4 weeks at doses of 540 mg, 360 mg, or 120 mg	Subcutaneous implantation of a gelatin hydrogel on days 1 and 15	No *in vivo* dosing regimen was described (the study was performed *in vitro* and structurally)
Mechanism of action	Simultaneous inhibition of both IL-31 and OSM signaling pathways	Blockade of OSMRβ, thereby simultaneously inhibiting both IL-31 and OSM signaling	Does not compete with ligand binding; instead, it inhibits signaling by inducing receptor internalization
Efficacy endpoints	Percentage change in WI-NRS at week 16; proportion of patients achieving a ≥4-point reduction in WI-NRS; proportion achieving a PN-IGA score of 0 or 1	Skin severity score, scratching behavior, and serum Th2 cytokines (IL-4, IL-13, IL-31, IgE)	Inhibition of STAT5 phosphorylation (IC_50_) and receptor internalisation assays
Observed efficacy	In the high-dose group, the change in WI-NRS was –56.2% versus –14.5% for placebo (p<0.001); 66.0% of patients achieved a ≥4-point reduction in WI-NRS; and 38.3% achieved a PN-IGA score of 0 or 1.	Significant reductions in skin severity score and scratching behavior; decreased serum levels of IL-4, IL-13, IL-31, and IgE.	202 IgG exhibited an IC_50_ of 0.93 nM, whereas its Fab fragment had an IC_50_ of 32 nM, representing an approximately 34-fold enhancement in potency for the bivalent form; it did not block cIL-31 binding.
References	(72)	(14)	(76)

## 6 Conclusion and outlook

Collectively, these findings highlight the therapeutic potential of targeting OSMRβ to modulate both pruritus and cutaneous inflammation; however, several important limitations must be considered (14, 76).

Nevertheless, several important limitations of the current evidence should be acknowledged. Available evidence on OSM/OSMRβ signaling is concentrated primarily in AD, psoriasis, PN, and FPLCA, and both the quantity and strength of evidence vary considerably among these disorders. For several disease-specific mechanisms, the evidence chain remains incomplete, with some conclusions relying predominantly on Level II animal studies or Level III *in vitro*/ex vivo experiments and lacking sufficient validation in Level I human tissues or clinical cohorts. Moreover, although OSMRβ is involved in both inflammatory and pruritic signaling, its potential contribution to other chronic pruritic dermatoses remains largely unexplored. Future studies should therefore broaden the spectrum of diseases investigated and determine whether OSM/OSMRβ signaling represents a shared pathogenic mechanism in other pruritic conditions, particularly those characterized by neuroimmune dysregulation, epidermal barrier dysfunction, or persistent cutaneous inflammation. Integrating human tissue studies with single-cell and spatial profiling, mechanistic experiments, longitudinal clinical cohorts, and therapeutic intervention studies will be important for defining disease-specific OSMRβ signaling patterns, clarifying the relative contributions and potential cross-regulation of the IL-31 and OSM axes, and identifying biomarkers that may predict response to OSMRβ-targeted therapy.

In summary, current evidence supports further investigation of OSMRβ as a potential therapeutic target at the intersection of cutaneous inflammation and pruritus. Further elucidation of its disease- and cell-specific functions, together with continued clinical evaluation of OSMRβ-directed therapies, will be essential to determine whether targeting this shared receptor can provide a broadly applicable therapeutic strategy across inflammatory and pruritic skin diseases (**Table 3**).

**Table 3:** Comparison of IL-31- and OSM-mediated OSMRβ signaling.

Feature	IL-31	OSM
Major cellular sources	Th2 cells, mast cells, macrophages (30, 57)	Activated T cells, monocytes/macrophages, dendritic cells, neutrophils (32, 82)
OSMRβ-containing receptor complex	IL-31RA/OSMRβ (29)	gp130/OSMRβ (type II OSM receptor) (18)
Major target cells	Sensory neurons, keratinocytes, fibroblasts, and immune cells (29, 30, 83)	Keratinocytes, fibroblasts, immune cells, sensory neurons (36, 37)
Shared downstream pathways	JAK/STAT, MAPK/ERK, PI3K/AKT	JAK/STAT, MAPK/ERK, PI3K/AKT
Representative signaling pattern	Predominant STAT3 activation in keratinocytes; MAPK/ERK and PI3K/AKT activation is cell-context dependent (29, 84)	Robust STAT3 and STAT1 activation in keratinocytes/fibroblasts; concurrent MAPK/ERK and PI3K/AKT signaling (39)
Sensory-neuron effect	Direct activation of IL-31RA-expressing pruriceptive sensory neurons, inducing itch (30)	Sensitization of OSMR-expressing sensory neurons rather than direct activation; enhanced responsiveness to pruritogens and neuronal excitability (34)
Major biological outcomes	Pruritus; regulation of keratinocyte function; cutaneous inflammation and barrier dysfunction; fibrosis	Cutaneous inflammation; keratinocyte activation and proliferation; tissue remodeling; sensory-neuron sensitization
Potential cross-regulation	IL-31 and OSM share OSMRβ as a common receptor subunit, providing a potential point of receptor-level interaction. OSM has been shown to upregulate OSMRβ expression in human epidermal keratinocytes; however, whether this directly alters IL-31 responsiveness remains to be established. Indirect cross-talk through changes in the inflammatory microenvironment is also plausible but remains incompletely defined (57).	
